# Understanding the Tumor Immune Microenvironment in Renal Cell Carcinoma

**DOI:** 10.3390/cancers15092500

**Published:** 2023-04-27

**Authors:** Daniel D. Shapiro, Brendan Dolan, Israa A. Laklouk, Sahar Rassi, Taja Lozar, Hamid Emamekhoo, Andrew L. Wentland, Meghan G. Lubner, Edwin Jason Abel

**Affiliations:** 1 Department of Urology, The University of Wisconsin School of Medicine and Public Health, Madison, WI 53726, USA; 2 Division of Urology, William S. Middleton Memorial Veterans Hospital, Madison, WI 53705, USA; 3 Department of Pathology, The University of Wisconsin School of Medicine and Public Health, Madison, WI 53705, USA; 4 McArdle Laboratory for Cancer Research, Department of Oncology, University of Wisconsin School of Medicine and Public Health, Madison, WI 53726, USA; 5 Department of Medical Oncology, The University of Wisconsin School of Medicine and Public Health, Madison, WI 53726, USA; 6 Department of Radiology, The University of Wisconsin School of Medicine and Public Health, Madison, WI 53726, USA

**Keywords:** renal cell carcinoma, immune microenvironment, tumor immunology, immune checkpoint inhibitor, immunotherapy

## Abstract

**Simple Summary:**

Over the last decade there has been a significant increase in the number of therapies that activate the host’s immune system to target and eliminate renal cell carcinoma (RCC) tumors. The superior efficacy of these agents demonstrates that host control of RCC requires a robust and effective immune response. Given the increasing incorporation of immune activating agents into routine clinical practice for the management of RCC, it is important for researchers and clinicians to understand the characteristics of the immune microenvironment in RCC tumors. The purpose of this review is to describe the concepts of the anti-tumor immune response to RCC and to provide a detailed summary of the current understanding of the immune response to RCC tumor development and progression. Additionally, this article explores how components of the immune microenvironment are being used to predict response to therapy and patient survival.

**Abstract:**

Scientific understanding of how the immune microenvironment interacts with renal cell carcinoma (RCC) has substantially increased over the last decade as a result of research investigations and applying immunotherapies, which modulate how the immune system targets and eliminates RCC tumor cells. Clinically, immune checkpoint inhibitor therapy (ICI) has revolutionized the treatment of advanced clear cell RCC because of improved outcomes compared to targeted molecular therapies. From an immunologic perspective, RCC is particularly interesting because tumors are known to be highly inflamed, but the mechanisms underlying the inflammation of the tumor immune microenvironment are atypical and not well described. While technological advances in gene sequencing and cellular imaging have enabled precise characterization of RCC immune cell phenotypes, multiple theories have been suggested regarding the functional significance of immune infiltration in RCC progression. The purpose of this review is to describe the general concepts of the anti-tumor immune response and to provide a detailed summary of the current understanding of the immune response to RCC tumor development and progression. This article describes immune cell phenotypes that have been reported in the RCC microenvironment and discusses the application of RCC immunophenotyping to predict response to ICI therapy and patient survival.

## 1. Introduction

In 2022, renal cell carcinoma (RCC) accounted for most of the estimated 79,000 new diagnoses of kidney cancer and 13,920 deaths, making it one of the most common malignancies in the United States [1,2]. For clinically localized or locally advanced disease, surgery is the primary treatment, but tumors with aggressive features, such as high tumor grade, presence of tumor thrombus, tumor necrosis and peritumoral fat invasion, may have higher rates of metastatic progression [3,4]. Consequently, RCC has been the focus of tumor microenvironment (TME) research to understand mechanisms of pathogenesis and to inform treatment approaches. Previously, angiogenic components of the TME in RCC emerged as promising targets, with improved survival demonstrated in metastatic RCC (mRCC) using tyrosine kinase inhibitors [5]. More recently, immune checkpoint inhibitors (ICI) have shown superior outcomes compared to tyrosine kinase inhibitor and mTOR inhibitor therapies in advanced RCC clinical trials [6,7,8,9]. Understanding the immunologic aspects of the RCC TME will be important to developing new therapeutic targets and prognostic signatures. Although ICIs have shown success for RCC treatment [10,11], our understanding of the mechanisms of anti-tumor immune response and immune dysregulation in RCC remains limited. This review aims to summarize our understanding of the basic concepts of RCC tumor-immune biology and discusses in detail the attempts to use components of the RCC immune microenvironment as prognostic and predictive biomarkers.

## 2. The Immune Response to Cancer

**Figure 1:**
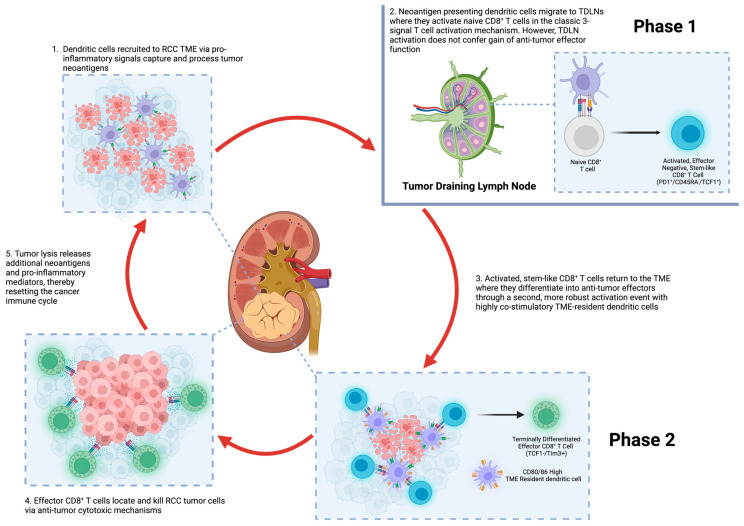
The renal cell carcinoma cancer immune cycle. Depiction of the immune response cycle to renal cell carcinoma. Dendritic cells (DCs) and other antigen presenting cells (APCs) are recruited to the tumor microenvironment where renal cell carcinoma (RCC) tumor neoantigens are captured, processed and then presented by DCs and APCs on MHC I and MHC II molecules (step 1). DCs and other APCs migrate to tumor draining lymph nodes (TDLNs), where they present tumor neoantigens to T cells (step 2). Naïve CD8^+^ T cells undergo a two-phase process to gain effector functions (as proposed by Prokhnevska et al. [16]). In phase 1, naïve CD8^+^ T cells become activated stem-like CD8^+^ T cells, which migrate to the tumor microenvironment and have the ability to replicate but do not yet possess effector functions. In phase 2, the stem-like CD8^+^ T cells differentiate into effector cells by a second co-stimulatory signal from tumor resident DCs. These terminally differentiated effector CD8^+^ T cells target and destroy tumor cells (step 4). This releases more RCC neoantigens, further potentiating the cancer immune cycle (step 5).

The tumor immune microenvironment is shaped by the response of the immune system to tumor cell development, which is distinct from the response to external pathogens. For example, recognition of “non-self” antigens on bacteria allows targeting and removal while avoiding autoimmunity. However, tumor cells are unique because non-synonymous somatic mutations of host genes produce “neoantigens” that may be recognized by immune cells as foreign, triggering a host immune response, which has been described as the “cancer-immunity cycle” (Figure 1) [12]. Tumor cell recognition is initiated primarily by dendritic cells (DCs), which act as the main antigen presenting cells (APCs) within the body. Neoantigens are captured and processed by DCs in the tumor microenvironment, which has migrated to the tumor under the influence of pro-inflammatory signals [13]. Certain types of mutations, such as frameshift mutations, may result in significantly altered peptide sequences leading to more robust immune responses [14]. Measures of tumor mutational burden (TMB) have been used as an indirect measure of neoantigen production and have been found to be associated with survival and improved response to ICI therapy in some tumors, such as melanoma and NSCLC; however, prior studies have suggested that TMB is less predictive of ICI success in RCC [14,15].

After neoantigen capture and processing by DCs and other APCs, cells migrate to tumor draining lymph nodes and present neoantigens to T cells, which become primed and activated leading to effector T cell responses. One critical step of the cancer-immunity cycle is the adaptive immune system recognizing that neoantigens are foreign and bypassing central tolerance. Activation of T cells triggers both effector and regulatory responses, and the balance of these two responses determines the overall tumor destruction. After activation, effector cells infiltrate into the tumor through specialized vascular endothelial cells with the aid of pro-inflammatory cytokines and chemokines. Accordingly, T cells recognize cancer cells expressing neoantigens loaded on MHC class I molecules with the T cell receptor (TCR) which ultimately results in killing the cancer cell [12,13]. CD8^+^ T cells release death-inducing granules containing granzymes and perforin which can create cancer cell membrane pores allowing enzyme entry and cell death [17]. Additionally, CD8^+^ can induce apoptosis by activating the FasL/Fas pathway, which activates caspases and endonucleases, leading to fragmentation of cancer cell DNA [17]. Tumor cell death releases more tumor neoantigens, which stimulates and reinforces the cancer-immune cycle. Acting as a negative balance or checkpoint, immune regulatory signals can dampen the host adaptive immune response and produce a pro-tumorigenic microenvironment. For example, expression of PD-L1 on tumor cells binds to PD-1 on CD8+ T cells resulting in reduced cytotoxicity, proliferation and response to TCR stimulation [13]. Inhibition of negative immune regulatory responses (by blocking PD-1 or CTLA-4 signaling) is the central tenant of ICI therapy and has been successful in improving immune destruction and clearance of multiple tumor types including RCC.

The recognition of tumor neoantigens and creation of an effector response within the tumor draining lymph nodes has been dogma and extrapolated from research investigating response to viral infections. More recent studies suggest that the adaptive immune response to tumors may be more complex. Prokhnevska et al. demonstrated a two-phase process in a mouse model of RCC [16]. First, CD8^+^ T cells are primed within the tumor draining lymph node. These stem-like CD8^+^ T cells (expressing TCF1 and PD-1) proliferate but do not express effector molecules associated with tumor cell killing. The stem-like CD8^+^ T cells then migrate to the tumor via chemokine signaling and express co-stimulatory receptors, maintaining a stem-like phenotype until a second phase of co-stimulation by APCs generates an effector transcriptional program necessary for tumor cell killing (Figure 1) [16]. This two phase effector T cell activation is thought to have implications for ICI therapy. Blocking PD-1 may promote activation and proliferation of stem-like CD8^+^ T cells, but a second stimulatory signal is required from APCs within the TME, which leads to an effector CD8^+^ T cell response and tumor killing. The necessity of a second stimulatory signal could help explain why there is significant variation in responses to anti-PD-1/PD-L1 therapies among different RCC tumors and between patients [16].

Heterogeneity and distribution of different immune cells within the TME is vast and varies significantly among different tumors [18]. In addition, the immune microenvironment composition within tumors changes as tumors evolve over time. More advanced tumors often develop a more immunosuppressive phenotype with decreased involvement of effector T cells [19,20]. For example, as colorectal tumors evolve from stage T1 to T4, T cell tumor infiltration significantly decreases [19]. In RCC, more advanced stages are associated with an increase of exhausted CD8+ T cell phenotypes and expression of an immunosuppressive M2 macrophage phenotype [20]. Although the distribution of immune cells within the TME has been frequently shown to be prognostic of clinical outcomes [18,21], it is critical to understand that tumor-immune interactions are dynamic and vary over time.

## 3. Cell Types of the Tumor Immune Microenvironment

The cancer microenvironment, including the immune microenvironment is a dynamic pathological ecosystem [22,23]. Several organs and tissues including the bone marrow, blood, spleen and tumor-draining lymph nodes form an interconnected immunological network to help create and deliver immune cells to the TME. The distribution of immune cell phenotypes within the TME influence tumor growth and patient outcomes. There are two main categories of immune cells: cells that comprise the innate and adaptive immune system. Broadly, components of the innate immune system include macrophages, dendritic cells, neutrophils, myeloid derived suppressor cells, natural killer (NK) cells, eosinophils, basophils, mast cells and innate lymphoid cells [24]. Components of the adaptive immune system include T cells (e.g., CD4^+^ and CD8^+^ T cells) and B cells. Many of these cell types have significant plasticity and can gain different phenotypes depending on the surrounding microenvironment signals and cellular interactions.

Macrophages are commonly observed immune cells in the RCC TME. These tumor associated macrophages (TAMs) have significant plasticity and vary between the M1 and M2 phenotypes. M1 cells have a tumor suppressive phenotype and express proinflammatory cytokines, which potentiate T_H_1 responses [24,25]. M2 macrophages tend to have a pro-tumorigenic, immunosuppressive phenotype [24]. Tumor cells can influence which phenotype a macrophage adopts by secreting IL-4 promoting the M2 phenotype, which has demonstrated the ability to remodel the surrounding tumor stroma, promoting tumor invasion [24,25]. Additionally, TAMs may repress cytotoxic T cells through a number of proposed mechanisms. These include depletion of L-arginine and reactive oxygen species generation, or they may modulate T cell function through expression of regulatory cytokines, such as IL-10 and TGF-β [26]. In general, RCC tumors with high infiltration of TAMs may limit anti-tumor T cell responses and are associated with a poor prognosis [27,28].

Dendritic cells are the primary innate immune cells that activate T cell mediated immune responses [29,30]. Dendritic cells are considered “professional antigen presenting cells” and uptake, process and present antigens through MHC class I and MHC class II molecules. Dendritic cells stimulate T cell activity through secretion of cytokines that induces T cell differentiation [30]. Other immune cells, such as B cells and macrophages, can also present antigens to T cells, but DCs are the most active T cell stimulating cells. Dendritic cells are broadly classified into classical DCs (cDCs), which can be further categorized as cDC1 and cDC2 subtypes, plasmacytoid DCs (pDCs) and monocyte-derived inflammatory DCs (moDCs) [31]. Similar to macrophages, the DC phenotype exists on a spectrum and DCs infiltrating tumors can be immunogenic or tumor tolerogenic. Tumors can also secrete cytokines to induce particular DC transcriptional and metabolic pathways that promote a tolerant environment such that DC will drive T_H_2 and T_reg_ responses, such as pathways that involve IDO, Arg1, iNOS, and STAT3 [24]. Given their phenotypic plasticity, the role of DCs in tumor control is variable. As with other cell types, the DC phenotype may be tumor stage dependent, and as tumors become more advanced, DCs may shift from a tumor suppressive to tumor promoting phenotype [24].

Another important member of the innate immune system is the natural killer (NK) cell. These innate lymphoid cells have cytotoxic effector functions [24,25]. Tumor cells are typically destroyed by CD8^+^ T cells, which rely on the expression of MHC class I molecule expression on the tumor cell surface for T cell recognition. Tumor cells can evade immune detection through the mutation or down regulation of MHC class I molecules. NK cells recognize and target cells that lack MHC class I expression in the TME as a mechanism to prevent immune evasion; however, NK cells are less efficient at destroying tumor cells compared to CD8^+^ T cells [13]. Gene expression data from The Cancer Genome Atlas (TCGA) revealed that ccRCC tumors had the highest probability of immune infiltration for several immune cell types, including NK cells compared to other tumor types [32]. Other studies using RCC patient-derived xenografts also demonstrate RNA signatures for inflamed RCC tumors, including tumors with heavy NK cell infiltration [33]. NK cells express a number of inhibitory immune checkpoint proteins similar to T cells including TIM-3, LAG-3 and TIGIT (T cell immunoglobulin and ITIM domain) as well as stimulatory checkpoints, such as 4-1BB, which may be potential targets for future therapeutic exploitation [34].

T cells are a major component of the adaptive immune response to RCC, which may determine how a tumor progresses over time [21]. Two major classes of T cells exist expressing either CD4 or CD8 cell surface proteins. CD4^+^ T “helper” cells can be divided into various phenotypic subtypes. T_H_1 cells are associated with an anti-tumor phenotype and help to stimulate a cytotoxic CD8^+^ T cell response via the production of IL-2 and IFNγ. Conversely, T_H_2 and T_reg_ CD4^+^ cells have more pro-tumorigenic phenotypes and lead to a reduced antitumor response through the production of inhibitory cytokines (e.g., IL-10, TGFβ and IL-35). T follicular helper (T_FH_) cells are another CD4^+^ subtype that express BCL-6, which have been found in tertiary lymphoid structures and interact with B cells to stimulate anti-tumor antibody production. T regulatory (T_reg_) cells express CD4, CD25 and FOXP3 [35], which primarily function to promote peripheral tolerance and prevent autoimmunity secondary to chronic T cell activation as seen in the TME [36]. These cells are hypothesized to help promote tumor growth by suppressing cytotoxic effector cells [35]. While these cells appear to have a pro-tumorigenic function, their presence has been associated with both a poor and favorable prognosis, as their presence may be indicative of an ongoing robust cytotoxic T cell response to the tumor [36]. T_reg_ cells within the TME have been demonstrated to promote loss of effector CD8^+^ T cell function through secretion of IL-35 leading to T cell exhaustion and upregulation of multiple inhibitory receptors (PD-1, TIM-3, LAG-3) on CD8^+^ T cells [37].

The primary anti-tumor effector cells are CD8^+^ T cells [38], which can be divided into different phenotypes based on differential gene expression. These phenotypes include naïve, effector, memory and exhausted CD8^+^ T cell phenotypes. Effector T cells are critical to anti-tumor immunity and express genes associated with cytotoxic activity, including *GZMA*, *GZMB* and *NKG7* [38]. Historically, it was believed that CD8^+^ T cells gain effector functions within tumor draining lymph nodes (TDLN), as is seen during viral infections, but recent work in RCC and prostate tumors demonstrates that some CD8^+^ T cells may undergo a two-phase activation process starting in the TDLN and ending with a second stimulatory signal in the TME [16]. CD8^+^ T cells recognize tumor cells through the T cell receptor (TCR) interacting with neoantigens loaded on MHC class I molecules present on the tumor cell surface.

Higher neoantigen load produced by homogenous tumor cells has been demonstrated to promote more effective immune surveillance in lung cancer. The investigators speculate that higher neoantigen heterogeneity may yield a lower antigen “dose”, which may not effectively stimulate an immune response [15]. This concept is relevant to RCC as these tumors tend to be highly heterogenous. Intratumoral heterogeneity may potentially result in a spatially heterogenous neoantigen load and a less effective CD8^+^ T cell response. Over time and as the tumor progresses, CD8^+^ T cells will begin to exhibit an “exhausted” phenotype within the TME. This appears to be a gradual change and not necessarily a distinct group of CD8^+^ cells. CD8^+^ T cells will begin to express various levels and combinations of inhibitory receptors, such as TIM-3, CTLA-4, LAG-3 and PD-1, and stimulation of these receptors leads to contraction of the immune response [25,38]. Modulation of the exhausted CD8^+^ T cell phenotype through inhibition of these receptors (such as PD-1 and CTLA-4 blockade) is the basis for ICI therapy and is the current first line systemic therapy for metastatic and adjuvant high risk ccRCC.

**Figure 2:**
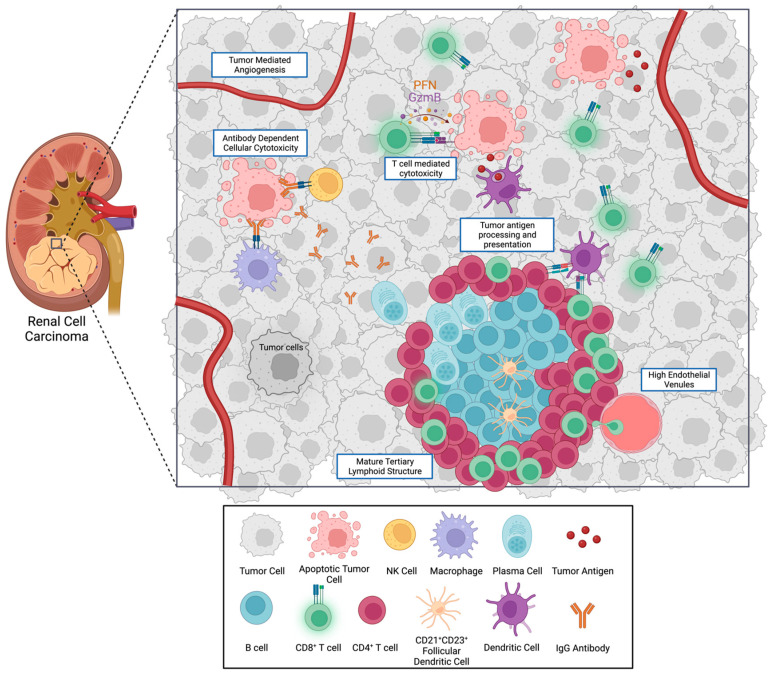
Components of the renal cell carcinoma tumor immune microenvironment. This figure demonstrates some of the many immune components of the tumor microenvironment. Mature tertiary lymphoid structures are composed of a germinal center containing follicular dendritic cells, B cells which differentiate into plasma cells secreting antibodies and a surrounding T cell zone composed of CD4^+^ and CD8^+^ T cells, which can be activated by antigen presenting cells. High endothelial venules (expressing PNAd) are also present within TLS, which are specialized capillaries that aid in lymphocyte migration. Plasma cells migrate from the TLS along fibroblast tracks via chemokine signaling [44] and continue to produce antibodies that bind to RCC tumor cells. This allows for antibody dependent cellular cytotoxicity (ADCC) when cells such as NK cells and macrophages recognize and destroy antibody bound tumor cells. Additionally, cytotoxic CD8^+^ T cells target tumor cells presenting neoantigens loaded on MHC class I molecules (i.e., T cell mediated cytotoxicity). Binding of these MHC class I molecules to the T cell receptor leads to the release of granzymes and perforin from the T cell, which creates pores in the tumor cell membrane and subsequent tumor cell death leading to further tumor neoantigen release.

Another critical member of the adaptive immune response to tumors are B cells, which can be subdivided into naïve, IgM memory, switched memory, germinal center, plasmablast, plasma cells and B regulatory (B_reg_) cells [39]. B cells localize in tumor draining lymph nodes and in ectopic lymphoid structures called tertiary lymphoid structures (TLS) in the TME [40]. Within TLS, B cells can undergo maturation from naïve B cells to memory B cells and plasma cells (Figure 2), which then propagate into the tumor bed. B cell phenotypes within the TME are highly heterogenous and B cells can exhibit both pro- and anti-tumorigenic functions. Anti-tumorigenic functions of B cells include the production of tumor specific antibodies and T cell priming and activation as well as direct cytotoxic activity. Conversely, B_reg_ cells can produce cytokines, such as IL-10, which inhibit cytotoxic CD8^+^ T cell activity [41]. B cells can also lead to immune tolerance and secrete cytokines that promote cancer cell growth [40]. In general, B cell infiltration has been correlated with improved clinical outcomes among multiple tumor types [41]; however, in ccRCC, higher expression of B cell associated genes was associated with worse prognosis based on analysis of TCGA gene expression data [42]. Additionally, increased B_reg_ infiltration has been associated with a poor prognosis in various tumor types [41,43].

## 4. Characteristics of the Renal Cell Carcinoma Tumor Immune Microenvironment

RCC is known to be one of the most highly immune infiltrated solid tumors containing a heterogeneous population of infiltrating immune cells [45,46]. Using the TCGA, a study by Rooney et al. used an RNA-based metric of immune cytolytic activity by measuring expression of granzyme A (*GZMA*) and perforin (*PRF1*), which are upregulated in activated CD8^+^ T cells. Among 18 different untreated primary tumor subtypes, ccRCC demonstrated the highest cytolytic activity of all tumors [47]. These findings were confirmed in a similar study that evaluated 19 different cancer types using the TCGA and demonstrated ccRCC and lung adenocarcinoma to have the highest T cell and overall immune infiltration gene signature score [48].

Different histologic subtypes of RCC have significantly different immune microenvironments, highlighting the tumor cell’s ability to generate or alter the immune response differently among the various RCC subtypes. Clear cell RCC has the highest degree of immune infiltration compared to either papillary or chromophobe RCC based on TCGA immune gene signature analyses [49]. Chromophobe RCC is associated with an increased T_H_17 gene signature; while IL-8 and CD56 NK cell gene signatures are increased in papillary RCC [49]. In an effort to identify a prognostic biomarker for RCC, Ricketts et al. identified that the T_H_2 gene signature, among various other immune cell gene signatures, was the only one correlating with poor survival among all RCC subtypes, including clear cell, papillary and chromophobe [49]. Conversely, the T_H_17 gene signature was associated with prolonged survival in ccRCC only [49].

Chevrier et al. conducted a detailed analysis of the RCC immune microenvironment by performing mass cytometry on 73 ccRCC tumors and 5 matched normal kidney samples, which provided a comprehensive “immune atlas” of RCC tumors [50]. Tumors within this study consisted of all grades and also included four patients with metastatic disease. The analysis demonstrated T cells to be the most common immune cell within the RCC immune microenvironment (composing 51% of immune cells), and myeloid cells (31%), NK cells (9%) and B cells (4%) were the next most common. Among T cells, various phenotypes were detected, including 11 different CD8^+^ T cell phenotypes and 8 CD4^+^ T cell phenotypes. Tumors demonstrated broad expression of PD-1 across T cell phenotypes; however, other inhibitory receptors, such as CTLA-4, 4-1BB and TIM-3, had more variable expression, which has implications related to therapeutic targeting of PD-1 versus other inhibitory molecules that may be involved with T cell exhaustion [50]. This study also characterized 17 different tumor associated macrophage (TAM) phenotypes, and demonstrated the transition between immature circulating monocytes to mature tissue macrophages. TAMs with high CD38 expression were associated with higher T_reg_ cells or exhausted T cells, which may indicate that CD38^+^ TAMs modulate T cell activity in the RCC TME. Importantly, different TAM populations demonstrated prognostic ability with clinical metrics [50].

Using paired single-cell RNA and T cell receptor sequencing in four ICI-naïve and two ICI-treated metastatic ccRCC patients, Krishna et al. dissected the RCC immune microenvironment, finding 5 CD8^+^ T cell phenotypic clusters, 5 CD4^+^ T cell clusters, 2 monocyte clusters, 3 dendritic cell clusters and 4 TAM clusters. Multiregional tumor sampling also demonstrated significant intratumoral immune diversity, with certain tumor regions with extensive T cell infiltration, while other portions were T cell excluded or TAM infiltrated. Interestingly, an ICI-resistant tumor was characterized by T cell exclusion and had the highest prevalence of TAM populations compared to all other tumors evaluated, raising the possibility that an immunosuppressive TAM and T cell exclusion phenotype may result in ICI resistance [27]. This study also demonstrated that tissue-resident CD8^+^ T cells (expressing CD69, ZNF683, CD103, PCD1 and LAG3) may promote anti-tumor immunity and appear to expand after ICI administration. The investigators speculate that tissue-resident T cells may swiftly recognize neoantigens and produce cytotoxic effector molecules, similar to their ability to rapidly respond to pathogenic infections [27].

Renal cell carcinoma tumors have significant TME spatial heterogeneity which impacts the density and phenotypes of immune cells across the tumor. Investigations of spatial heterogeneity have demonstrated that the degree of T cell exhaustion strongly correlates with the cell’s location within the tumor. The physical location of the immune cells may drive T cell receptor clonotype enrichment to a greater extent than the tumor’s somatic mutation heterogeneity [51]. A study by Li et al. demonstrated that T cell clonotypes were enriched in only certain regions across the tumor, and these regions had negligible differences in somatic mutations [51]. Additionally, particular TAM phenotypes appear to enrich in either the tumor core or invasive front. The study also demonstrated that T cell clones appear to infiltrate tumors and undergo a gradual phenotypic transformation from activation to dysfunction. Taken together, these data suggest that sampling a single tumor region will unlikely reflect the extent of T cell (i.e., T cell receptor) clonal expansion across the entire tumor. The study also demonstrated that the location of the tumor and immune cell interaction impacts the ability of the tumor to progress. Renal cell carcinoma cells expressing an epithelial-mesenchymal transition (EMT) gene program, which is critical for metastatic competence, were more abundant at the tumor/normal tissue interface. These EMT-high RCC cells colocalized with IL-1β expressing macrophages, which can break down the surrounding collagen-rich stroma. This suggests that macrophages may help to promote tumor invasion [51].

How cells interact and organize in the TME is important for an effective immune response. Tertiary lymphoid structures (TLSs) have been increasingly recognized as possible sites of tumor antigen recognition and generation of adaptive immune responses locally within the TME [44,52,53,54,55,56,57,58,59,60,61,62,63,64]. Tertiary lymphoid structures are organized aggregates of immune cells that arise, unlike lymph nodes, postnatally in non-lymphoid tissues [62]. The organization of TLS consists of an inner zone of CD20^+^ B cells surrounded by T cells, mimicking lymph follicles of secondary lymphoid organs (Figure 2) [55,62]. T cells within TLSs consist of CD4^+^ T_FH_, T_H_1, T_regs_ and CD8^+^ cytotoxic T cells. CD21^+^CD23^+^ follicular dendritic cells are also present. The TLS is surrounded by specialized blood vessels called high endothelial venules (HEVs) that promote lymphocyte extravasation into the TME [54]. Tertiary lymphoid structures appear to have the ability to generate adaptive immune responses to tumor cells independent of more distant tumor draining lymph nodes. Mice lacking secondary lymphoid organs can still generate antitumor immune responses including effective T cell priming within intratumor TLS [55]. In RCC specifically, spatial transcriptomic analyses of primary tumor samples have shown TLSs to be sites of plasma cell maturation, which then migrate from TLS into the nearby TME guided by local fibroblast chemokines [44]. The plasma cells secrete IgG antibody which then binds and induces tumor cell apoptosis, possibly through a macrophage induced mechanism as evidenced by the higher infiltration of CD68+ macrophages surrounding IgG bound tumor cells [44]. Tertiary lymphoid structures and B cells localized within TLSs also appear to promote ICI response. Higher expression of B cell related genes was shown to be present in metastatic RCC tumors responsive to ICI. These intratumoral B cells were shown to localize within TLSs, and patients with higher TLS density were more likely to respond to ICI therapy [64].

One common research strategy is to categorize the intensity and type of immune responses within individual RCC tumors to describe the spectrum of immune responses. The phenotypes typically range from tumors that have a severely limited or absent immune infiltration to tumors that are heavily infiltrated with immune cells. Table 1 lists tumor immune microenvironment classification systems and the relative prognosis associated with each class. The classifications often not only incorporate the quantity of immune cell infiltration but also the degree of activation of individual immune cells. For example, Clark et al. characterized the immune infiltration using transcriptomic profiles into four categories: (1) CD8^+^ inflamed tumors which had high CD8^+^ infiltration with an effector gene signature characterized by increased IFNγ signaling; (2) CD8^-^ inflamed tumors characterized by an innate immune signature with increased dendritic and macrophage cells in the TME; (3) VEGF immune desert tumors with a high stromal gene signature, increased endothelial cells and enrichment of an angiogenesis signature; and (4) metabolic immune desert tumors, which had low immune and stromal scores as well as increased mTOR signaling and a unique metabolic profile. The lack of immune cells in the metabolic immune desert tumors suggests that a hypoxic, nutrient poor microenvironment may be immunosuppressive [65]. This demonstrates just one of many classification systems, which in general demonstrate a spectrum of immune infiltration, and no consensus has been reached as to which classification system is most prognostic or predictive of systemic therapy response. These classification systems also suggest an evolution of the immune response to tumors over time. Further studies have investigated how the immune response evolves as tumors progress.

### 4.1. Immune Microenvironment Changes during Tumor Development and Treatment

The immune microenvironment is dynamic, and the phenotype of immune cells within the tumor changes over the course of tumor development. Recent investigations have focused on how the immune cell phenotypes alter over the course of tumor development [34,51,69]. Braun et al. evaluated how the immune cell phenotypes change from early to advanced (i.e., metastatic) disease stages. The investigators profiled immune cells from ccRCC tumors using single-cell transcriptomics from 13 patients with advancing disease stage [20]. The study demonstrated within T cell populations, there was marked transcriptional heterogeneity consisting of 19 individual populations of T cells including T_reg_ cells, CD4^+^ with activated or central memory phenotypes, CD8^+^ tissue resident memory cells, and a large population of exhausted CD8^+^ T cells. Six of the CD8^+^ populations expressed markers of exhaustion such as PD-1, *TOX* and TIM-3 at high or moderate levels, indicating a terminally exhausted phenotype. Another group of CD8^+^ T cells expressed cytotoxic molecules consistent with an effector population. A group of three CD8^+^ clusters expressed *ZNF683* (HOBIT), *PRDM1* (BLIMP-1) and *ITGAE* (CD103), indicative of a tissue-resident memory phenotype. Among the CD4^+^ T cell population, 3 broad phenotypes were identified including an activated, central memory and T_reg_ population. Among early stage ccRCC tumors, effector CD8^+^ T cells were enriched while metastatic ccRCC tumors were enriched for terminally exhausted CD8^+^ T cells. With advancing disease stage, there was an increase in expression of inhibitory checkpoints such as PD-1, TIM-3 and LAG-3. Markers of progenitor or stem-like T cells, including TCF1 and T-bet, were increased early but decreased with disease progression. Overall, these data demonstrate a progressive T cell exhaustion with advancing disease stage. Tumors begin with an initial infiltration of cytotoxic CD8^+^ T cells followed by progressive immune dysfunction leading to terminally exhausted CD8^+^ T cells in advanced disease stages [20]. Interestingly, terminally exhausted CD8^+^ T cells were highly clonal (based on TCR sequencing) and represented a high proportion of the total number of T cells within the TME, suggesting a possible limited number of neoantigens are being targeted by T cells [20]. The findings by Braun et al. were confirmed by another study demonstrating that T cell clones entering ccRCC tumors undergo a phenotypic transition over time from an effector state to progressive dysfunction and ultimately terminal exhaustion with advancing disease stage [51].

Tumor associated macrophages (TAMs) also shift phenotypically with advancing disease stage. Early-stage tumors contain TAMs expressing more proinflammatory genes; however, TAMs shift to an anti-inflammatory gene signature with advancing disease. Additionally, the expression of M2 genes, which are associated with a pro-tumorigenic microenvironment, increases with metastatic disease [20]. M2 TAMs interact with CD8^+^ T cells through expression of multiple T cell immune checkpoint ligands, including PD-L1, CD80 and CD86 (which bind CTLA-4), CD155 (binds TIGIT) and Galectin-9 (binds TIM-3), and these interactions are associated with a worse overall survival [20]. Modulation of terminally exhausted T cells by inhibiting molecules, such as PD-1, PD-L1 and CTLA-4, is the basis for ICI therapy, which has led to significant improvements in the survival of patients with advanced RCC.

The immune microenvironment additionally shifts in response to ICI therapy. Bi et al. investigated immune microenvironment changes before and after ICI therapy, using single-cell transcriptomic dissection of mRCC tumor biopsies [70]. TAMs in responders to ICI therapy shift to a pro-inflammatory state, possibly secondary to IFNγ, produced by CD8^+^ T cells. Also, CD8^+^ T cells express higher levels of co-inhibitory receptors and effector molecules such as GZMB, PRF1 and IFNG [70]. Despite this significantly more inflammatory immune microenvironment in responders to ICI therapy, these tumors also had a profound increase in CD8^+^ T cell checkpoint genes and TAM anti-inflammatory signaling genes, suggesting a possible route to eventual ICI resistance [70].

### 4.2. Renal Cell Carcinoma Tumor Antigens and Genomic Correlations with Immune Response

As discussed, tumor neoantigens are critical components for immune cell recognition. Tumor mutational burden (TMB) is often used as a surrogate marker for neoantigen production and has been predictive of ICI responsiveness and degree of immune infiltration in multiple tumor types. However, RCC tumors only demonstrate a relatively moderate TMB, which has not been reliably predictive of RCC response to ICI therapy [34,71,72]. Compared to melanoma and non-small cell lung cancer, which harbor 10–400 mutations/megabase, ccRCC only harbors about 1.1 mutation/megabase. Although measured TMB is lower, RCC still demonstrates one of the highest levels of immune infiltration, particularly T cell infiltration, in the TME compared to other tumor types [71]. One possible explanation for this may be how TMB has been previously quantified, with the most common method being measurement of single nucleotide variants (SNVs). However, SNVs may not always produce inflammatory neoantigens which might confound TMB data for RCC. Mutations caused by insertions and deletions (“indels”) are much more likely to cause frameshifts leading to altered protein structures and formation of neoantigens that are recognized by immune cells. While RCC may have lower TMB, indels are highly abundant in RCC, which may lead to a higher neoantigen load and immune infiltration [73,74], despite relatively low measured SNV.

Beyond TMB, investigations have been performed linking particular RCC genotypes to the degree of tumor immune infiltration. The central pathway of tumor development in ccRCC is caused by the loss or inactivation of the tumor suppressor VHL located on chromosome 3p. Loss of VHL is often associated with loss of other tumor suppressor genes including PBRM1, BAP1 and SETD2, which are also located on chromosome 3p [75]. VHL inactivation causes stabilization of hypoxia inducible factors, HIF-1α and HIF-2α [75,76]. HIF activation causes expression of genes regulating angiogenesis, glycolysis and apoptosis [75]. HIF proteins also impact the tumor immune microenvironment [77]. In an immune competent murine autochthonous model of ccRCC, *Hif2a* deletion led to increased expression of immune infiltration mRNA signatures, antigen presentation and interferon activity. Additionally, greater effector CD8^+^ T cell infiltration (expressing markers of CD69 and perforin) was detected. In general, mice with *HIF2a* deletions developed less tumors compared to *HIF1a* mutants, which may be related to greater anti-tumor immune response secondary to *HIF2a* deletion [77]. In summary, activation of HIF-2α in ccRCC may act as a suppressor of T cell inflammation [77,78].

Mutations in *PBRM1* and *BAP1* are also critical driver events of ccRCC tumor development and are known to be prognostic for outcomes in RCC [75]. Studies have investigated their impact on the immune microenvironment. A cohort of metastatic ccRCC patients had whole exome sequencing (WES) of tumor tissue. Tumors harboring *PBRM1* loss of function (LOF) mutations were more likely to respond to ICI therapy [71]. Additionally, *PBRM1* LOF mutations were associated with lower expression of immune-inhibitory ligands within the tumor [71]. A separate study demonstrated that mRCC primary tumors containing truncating *PBRM1* mutations were less immune infiltrated overall and had a lower total CD8^+^ T cell infiltration compared to tumors with intact *PBRM1* [68]. Loss of PBRM1 function causes a reduction in IFNγ-STAT1 signaling in both murine and human RCC cell lines; thus, PBRM1 inactivation leads to a less immunogenic TME [79]. Conversely, tumors harboring *BAP1* mutations have been associated with heavy CD8^+^ T cell inflammation [33,65].

### 4.3. The Predictive and Prognostic Capability of the Tumor Immune Microenvironment in RCC

One goal of immune microenvironment research has been to identify immune based biomarkers that have the ability to prognosticate patient survival as well as predict response to ICI or anti-angiogenic therapies. Multiple investigations have identified potential biomarkers with occasionally conflicting results [32,34,48,49,65,66,67,68,69,73,80,81,82,83]. Table 2 lists some immune based biomarkers evaluated in multiple studies [84]. Studies have been heterogeneous in terms of the stage of tumors evaluated (early versus advanced stage), number of samples taken from individual tumors, whether the primary or metastatic tumor was sampled, and differences in how the immune microenvironment was evaluated (based on bulk or single cell sequencing signatures, immunofluorescence, biopsies versus whole slides, etc.). These differences make comparisons of potential biomarkers among studies difficult but there are some consistent themes within the available data.

Multiple studies have evaluated the immune microenvironment’s relation to patient prognosis. Renal cell carcinoma tumors that are heavily infiltrated with T cells have been associated with a worse prognosis compared to many other solid tumors in which T cell infiltration is often associated with a favorable prognosis [32,34,48]. Clear cell RCC tumors that are heavily infiltrated with T cells as measured by gene-based signatures are correlated with higher stage and grade as well as worse cancer specific survival [48]. Clark et al. demonstrated across a range of ccRCC tumor stages, from localized to metastatic, tumors classified as CD8^+^ inflamed, were associated with worse overall survival and other poor prognostic features, including higher *BAP1* mutations, higher grade tumors and increased *PD-1/PD-L1* expression [65]. Similarly, Giraldo et al. evaluated the immune microenvironment of metastatic ccRCC tumors and demonstrated patients with high CD8^+^ infiltration had about a twofold increase in risk of mortality compared to tumors with low CD8^+^ infiltration [82]. Another study of localized ccRCC tumors demonstrated that the ratio between effector T cell to T_reg_ cells was positively correlated with a lower rate of recurrence in localized ccRCC tumors [83]. An exhaustive phenotypic characterization of immune cells within localized ccRCC tumors using flow cytometric analysis showed that tumors at high risk of early progression contained a population of CD8^+^ PD-1^+^ cells that co-expressed TIM-3 and LAG-3 and exhibited an exhausted phenotype with decreased cytotoxic potential, polyclonality and enrichment of M2 TAMs [66]. These tumors, termed “immune regulated”, may be ideal for post-nephrectomy adjuvant ICI therapy [66,86]. Thus, not only the density of particular immune cells, for example CD8^+^ T cells, but also their phenotype (i.e., activated, exhausted) is critical in determining their prognostic capability. Currently, no immune-based gene signatures or biomarkers have been identified for routine clinical use to improve clinical decision making.

In addition to prognosis, studies have evaluated the ability of the immune microenvironment to predict response to systemic therapies for RCC, most often in the metastatic setting. A phase II trial, IMmotion150, evaluated anti-PD-L1 therapy (atezolizumab) with or without an anti-VEGF antibody (bevacizumab) compared to the tyrosine kinase inhibitor, sunitinib. One theory is that VEGF, which is upregulated in ccRCC, may enhance cancer immune evasion and blocking VEGF with bevacizumab may improve the immune activation triggered by atezolizumab [86]. As part of this trial, investigators evaluated gene signatures of the TME and how they correlated with clinical outcomes. The trial demonstrated the addition of bevacizumab to atezolizumab improved progression free survival compared to either atezolizumab or sunitinib alone among tumors with ≥1% PD-L1 positivity [80]. Among tumors with a high T_effector_ gene signature (defined by increased expression of *CD8A*, *EOMES*, *PRF1*, *IFNG*, *CD274*), the combination of atezolizumab + bevacizumab had significantly improved progression free survival compared to sunitinib monotherapy. This was further confirmed by the phase III IMmotion151 trial, which again demonstrated that tumors with a T_effector_ signature had an improved objective response rate and progression free survival when treated with atezolizumab + bevacizumab compared to sunitinib [87]. A study by Hakimi et al. evaluated 409 tumors by unsupervised clustering of microarray data from patients with metastatic ccRCC treated with either pazopanib or sunitinib as part of the COMPARZ trial [67]. The study identified 4 biologically distinct molecular “clusters” with different therapeutic responses. Cluster 4 had the worst overall and progression free survival probability, and this cluster was associated with less frequent *PBRM1* mutations as well as enrichment for *TP53* and *BAP1* mutations. In regard to the immune microenvironment, cluster 4 had significant enrichment for inflammatory signatures including IFNγ gene signatures as well as the highest total immune infiltrate of the 4 clusters. Immune deconvolution analysis demonstrated that macrophages were the dominant immune population within this cluster. Macrophage infiltration, particularly the M2 phenotype, was associated with worse overall survival among all patients. When determining response to TKI therapy, patients with a high angiogenesis gene signature and low macrophage infiltration had the best response to TKI therapy, thus serving as a predictive biomarker among patients within this trial but having not been further validated [67].

Carlisle et al. evaluated how T cell response in RCC was affected by ICI therapy and how a patient’s preexisting immune response to RCC may correlate with response to ICI therapy [81]. Investigators collected peripheral blood before and after ICI therapy administration in 36 mRCC patients (27 clear cell and 9 non-clear cell patients). Patients with increased expansion of HLA-DR^+^CD38^+^CD8^+^ T cells had the largest reductions in tumor size and longest progression free survival. This T cell phenotype expressed high levels of cytotoxic effector genes, including perforin-1, GZMB and IFNγ. Additional immunofluorescence studies of the primary tumors demonstrated that tumors harboring greater infiltration of CD8^+^ T cells, more TCF-1^+^CD8^+^ T cells and more MHCII^+^ cells were more likely to benefit from ICI therapy [81]. In regard to TCF-1^+^CD8^+^ T cells, a prior study had demonstrated that kidney tumors harbor these cells in areas of APCs. TCF-1^+^CD8^+^ T cells have stem-like properties, allowing them to produce more effector CD8^+^ T cells [88]. The presence of these intra-tumoral niches predict the magnitude of T cell infiltration and patient survival. Tumors that progress appear to lose these intra-tumoral niches, suggesting a possible mechanism of tumor immune escape [88]. Overall, similar to biomarkers for prognostication, a definitive, clinically useful predictive immune based biomarker has yet to be developed.

## 5. Future Directions

As further knowledge is gained exploring the tumor immune microenvironment, this may lead to successful new therapies exploiting the tumor-immune interaction, including treatments targeting tumor specific antigens, multi-antigen vaccines, personalized peptide vaccines and engineered T cell therapies [89]. Future therapies to overcome the inhibitory immune checkpoints beyond CTLA-4 and PD-1/PD-L1, such as therapies targeting LAG-3, TIM-3 and TIGIT, may be clinically beneficial since immune exhaustion and subsequent tumor escape remains critical for RCC progression. Additionally, investigations are underway to target immune metabolic pathways and stimulatory checkpoints (4-1BB and OX40), which may enhance immunotherapy responses [34].

Another potentially impactful factor shaping the immune response to renal cell carcinoma is the host microbiome. Numerous studies have shown that the gut microbiome influences the immune response to tumors, and therapeutic responses may be improved via its modulation [90,91,92,93]. Specific bacteria can stimulate the production of pro-inflammatory cytokines or anti-inflammatory cytokines. The byproducts of microbial metabolism can act as carcinogens. Also, the microbiome can educate immune cells, thus determining an individual’s overall immune response [94]. A recent phase 1 randomized trial by Dizman et al. randomized 30 treatment-naïve patients with mRCC to nivolumab and ipilimumab, with or without daily oral CBM588. CBM588 is a bifidogenic live bacterial product. Progression free survival was significantly longer in patients receiving ICI therapy with CBM588 (12.7 vs. 2.5 months, HR 0.15 95% CI 0.05–0.47, *p* = 0.001) with no significant difference in toxicity between study arms [95]. These data reinforce similar findings from patients with non-small cell lung cancer receiving CBM588. Possible mechanisms include increased propionate, a short chain fatty acid that has anti-tumor properties. The microbiota may upregulate chemokines, such as those involved in dendritic and cytotoxic T cell recruitment. These findings warrant further validation. Continued investigation of the host microbiome will likely help develop therapies to augment current and future immune activating agents.

It is necessary to explore a variety of clinical and behavioral variables that may impact tumorigenesis, immune response and treatment outcomes. For example, one interesting area of research includes the role of the circadian rhythm in the immune response to RCC. Clock genes, such as *CLOCK*, *BMAL1*, *PER* and *CRY,* regulate many processes, including cell division and metabolism [96]. Some studies have shown that alteration of these genes is linked to poor prognosis in RCC. Additionally, the circadian rhythm can influence the RCC immune microenvironment, which can impact tumor surveillance and response to therapy [96].

A possible future experimental roadmap to understand the immune microenvironment in RCC will likely include the following components: comprehensive molecular profiling including single-cell RNA sequencing and spatial transcriptomics/proteomics to identify different immune cell populations and activation status, validation of these populations through flow cytometry and immunohistochemistry, functional evaluation of immune cell populations both in vitro and in vivo, immune checkpoint analysis to determine the expression of checkpoint molecules in RCC tumor samples and their association with patient outcomes and tumor infiltrating lymphocyte isolation and characterization with T cell receptor sequencing to assess clonality and antigen specificity. Additionally, investigators should perform preclinical testing of novel immunotherapies developed from the insights gained from the prior steps. This should be followed by clinical translation of these novel immunotherapies to patients to generate new or synergistic therapies that improve patient survival and quality of life. Finally, studying long term outcomes of current and new therapies will help develop biomarkers for treatment response.

## 6. Conclusions

Renal cell carcinoma is one of the most highly immune infiltrated tumors and often responds to ICI therapy. Increasing our understanding of the RCC tumor immune microenvironment is critical for future progress [89]. Immune based biomarkers that are prognostic and predictive are continually being investigated. Tissue heterogeneity significantly limits the development of broadly applicable biomarkers, and further efforts to understand the intra- and inter-tumoral immune microenvironment heterogeneity are underway. Future studies should focus on other RCC histologic subtypes as the majority of studies to date have been conducted in the clear cell subtype. Finally, research should continue to investigate the impact of localized therapy, such as surgery, ablation and radiation on the tumor-immune interaction, and how these therapies can be effectively combined with systemic therapies to understand the optimal multidisciplinary approach to RCC treatment that improves patient survival and quality of life.

## Figures and Tables

**Table 1:** Tumor microenvironment classification systems.

Study	RCC Subtype	Stages Studied	Method of Categorization	Tumor Microenvironment Categorization	Category Characteristics	Category Prognosis
Senbabaoglu et al., 2016 [48]	Clear cell	All Stages	TCGA based gene expression signatures	Non-infiltrated	Low T cell infiltration, low stromal score, increased metabolism and mitochondrial related genes	Best
				Heterogeneously Infiltrated	Increased angiogenesis-related gene expression, c-KIT and Smad1	Intermediate
				T cell Enriched	High T cell infiltration, high granzyme B and IFNγ expression	Worst
Giraldo et al., 2017 [66]	Clear cell	Localized RCC	Multiparametric flow cytometric immunophenotypic analysis	Immune activated	Increased CD8^+^ clonality, increased cytotoxic genes	Best
				Immune silent	Low levels tumor infiltrating lymphocytes	Intermediate
				Immune regulated	M2-rich, poorly cytotoxic, increased T_reg_	Worst
Clark et al., 2019 [65]	Clear cell	All Stages	Transcriptomic and proteomic microenvironment signatures	VEGF immune desert	Elevated stromal score, endothelial enrichment	Best
				CD8^-^ Inflamed	Innate immune signature, fibroblast signature	Intermediate
				Metabolic immune desert	Low immune and stromal scores, elevated mitochondrial, OXPHOS, glycolysis protein expression	Intermediate
				CD8^+^ inflamed	High CD8^+^ infiltration, *BAP1* mutations, CD38 expression, IFNγ signaling	Worst
Hakimi et al., 2019 [67]	Clear cell	Metastatic	Somatic mutation analysis, RNA and protein expression	Cluster 3	High *PBRM1* mutations, high angiogenesis score, moderate immune infiltration	Best
				Cluster 2	Moderate angiogenesis score, moderate immune infiltration	Intermediate
				Cluster 1	Lowest angiogenesis score, lowest immune infiltration	Intermediate
				Cluster 4	High *BAP1* mutation, moderate angiogenesis score, high immune infiltration, higher PD-L1 expression	Worst
Braun et al., 2020 [68]	Clear cell	Metastatic	Integrated genetic, transcriptomic and immunopathologic analysis	Immune Excluded	5-fold more CD8^+^ T cells at the tumor margin than in tumor center	No difference in prognosis
				Immune Desert	Not excluded and below 25th percentile for CD8^+^ T cells, 50 cells/mm^2^ in the tumor center	
				Immune Infiltrated	Not excluded and ≥25th percentile for CD8^+^ T cells in the tumor center. Enriched for M1 macrophages, CD4^+^ memory T cells, NK cells	

**Table 2:** Renal cell carcinoma immune based biomarkers.

Biomarker	Description	Investigational Use	Limitations
PD-L1 [6,10,84]	Cell surface protein	Prognostic biomarker Predictive biomarker for ICI response	Studies failed to show utility as either prognostic or predictive Tumor spatial heterogeneity, lack multi-region sampling Cellular expression heterogeneity Lack of standardized assay
Tumor Mutational Burden [80]	Number of non-synonymous somatic mutations in tumor DNA	Predictive biomarker for ICI response	Studies failed to show predictive capacity RCC has low TMB Lack of standardized assays
Tumor Neoantigen Burden [80]	Number of immunogenic tumor-specific proteins (neoantigens) predicted by quantity of somatic mutations	Predictive biomarker for ICI response	Studies failed to show predictive capacity Lack of standardized predictive algorithms to determine neoantigen load
Renal 101 Immuno signature [85]	26-gene immune based signature	Prognostic of progression free survival in setting of ICI therapy	Has not been used outside of clinical trial setting
T-effector score [80]	Five gene signature (CD8A, EOMES, PRF1, IFNG, CD274)	Prognostic of progression free survival in setting of ICI + anti-VEGF therapy	Has not been used outside of clinical trial setting
Myeloid inflammation [80]	Six gene signature (IL-6, CXCL1, CXCL2, CXCL3, CXCL8, PTGS2)	Prognostic of progression free survival in setting of ICI + anti-VEGF therapy	Has not been used outside of clinical trial setting

## References

[1] Seer Cancer Stat Facts: Kidney and Renal Pelvis Cancer. National Cancer Institute: Bethesda, MD, USA, 2022. https://seer.Cancer.Gov/statfacts/html/kidrp.Html.

[2] Siegel R.L., Miller K.D., Fuchs H.E., Jemal A. (2022). Cancer statistics, 2022. CA A Cancer J. Clin..

[3] Mattila K.E., Laajala T.D., Tornberg S.V., Kilpeläinen T.P., Vainio P. (2021). A three-feature prediction model for metastasis-free survival after surgery of localized clear cell renal cell carcinoma. Sci. Rep..

[4] Leibovich B.C., Blute M.L., Cheville J.C., Lohse C.M., Frank I., Kwon E.D., Weaver A.L., Parker A.S., Zincke H. (2003). Prediction of progression after radical nephrectomy for patients with clear cell renal cell carcinoma: A stratification tool for prospective clinical trials. Cancer.

[5] Motzer R.J., Hutson T.E., Tomczak P., Michaelson M.D., Bukowski R.M., Oudard S., Negrier S., Szczylik C., Pili R., Bjarnason G.A. (2009). Overall Survival and Updated Results for Sunitinib Compared with Interferon Alfa in Patients With Metastatic Renal Cell Carcinoma. J. Clin. Oncol..

[6] Motzer R.J., Tannir N.M., McDermott D.F., Aren Frontera O., Melichar B., Choueiri T.K., Plimack E.R., Barthélémy P., Porta C., George S. (2018). Nivolumab plus Ipilimumab versus Sunitinib in Advanced Renal-Cell Carcinoma. N. Engl. J. Med..

[7] Rini B.I., Plimack E.R., Stus V., Gafanov R., Hawkins R., Nosov D., Pouliot F., Alekseev B., Soulières D., Melichar B. (2019). Pembrolizumab plus Axitinib versus Sunitinib for Advanced Renal-Cell Carcinoma. N. Engl. J. Med..

[8] Motzer R.J., Rini B.I., McDermott D.F., Aren Frontera O., Hammers H.J., Carducci M.A., Salman P., Escudier B., Beuselinck B., Amin A. (2019). Nivolumab plus ipilimumab versus sunitinib in first-line treatment for advanced renal cell carcinoma: Extended follow-up of efficacy and safety results from a randomised, controlled, phase 3 trial. Lancet Oncol..

[9] Motzer R.J., Escudier B., George S., Hammers H.J., Srinivas S., Tykodi S.S., Sosman J.A., Plimack E.R., Procopio G., McDermott D.F. (2020). Nivolumab versus everolimus in patients with advanced renal cell carcinoma: Updated results with long-term follow-up of the randomized, open-label, phase 3 checkmate 025 trial. Cancer.

[10] Choueiri T.K., Tomczak P., Park S.H., Venugopal B., Ferguson T., Chang Y.-H., Hajek J., Symeonides S.N., Lee J.L., Sarwar N. (2021). Adjuvant Pembrolizumab after Nephrectomy in Renal-Cell Carcinoma. N. Engl. J. Med..

[11] Powles T., Tomczak P., Park S.H., Venugopal B., Ferguson T., Symeonides S.N., Hajek J., Gurney H., Chang Y.-H., Lee J.L. (2022). Pembrolizumab versus placebo as post-nephrectomy adjuvant therapy for clear cell renal cell carcinoma (KEYNOTE-564): 30-month follow-up analysis of a multicentre, randomised, double-blind, placebo-controlled, phase 3 trial. Lancet Oncol..

[12] Chen D.S., Mellman I. (2013). Oncology Meets Immunology: The Cancer-Immunity Cycle. Immunity.

[13] Becht E., Giraldo N.A., Germain C., de Reyniès A., Laurent-Puig P., Zucman-Rossi J., Dieu-Nosjean M.-C., Sautès-Fridman C., Fridman W.H. (2016). Immune Contexture, Immunoscore, and Malignant Cell Molecular Subgroups for Prognostic and Theranostic Classifications of Cancers. Adv. Immunol..

[14] DiNatale R.G., Hakimi A.A., Chan T.A. (2020). Genomics-based immuno-oncology: Bridging the gap between immunology and tumor biology. Hum. Mol. Genet..

[15] McGranahan N., Furness A.J.S., Rosenthal R., Ramskov S., Lyngaa R., Saini S.K., Jamal-Hanjani M., Wilson G.A., Birkbak N.J., Hiley C.T. (2016). Clonal neoantigens elicit T cell immunoreactivity and sensitivity to immune checkpoint blockade. Science.

[16] Prokhnevska N., Cardenas M.A., Valanparambil R.M., Sobierajska E., Barwick B.G., Jansen C., Moon A.R., Gregorova P., Delbalzo L., Greenwald R. (2022). CD8+ T cell activation in cancer comprises an initial activation phase in lymph nodes followed by effector differentiation within the tumor. Immunity.

[17] Raskov H., Orhan A., Christensen J.P., Gögenur I. (2021). Cytotoxic CD8+ T cells in cancer and cancer immunotherapy. Br. J. Cancer.

[18] Fridman W.H., Pagès F., Sautès-Fridman C., Galon J. (2012). The immune contexture in human tumours: Impact on clinical outcome. Nat. Rev. Cancer.

[19] Bindea G., Mlecnik B., Tosolini M., Kirilovsky A., Waldner M., Obenauf A.C., Angell H., Fredriksen T., Lafontaine L., Berger A. (2013). Spatiotemporal Dynamics of Intratumoral Immune Cells Reveal the Immune Landscape in Human Cancer. Immunity.

[20] Braun D.A., Street K., Burke K.P., Cookmeyer D.L., Denize T., Pedersen C.B., Gohil S.H., Schindler N., Pomerance L., Hirsch L. (2021). Progressive immune dysfunction with advancing disease stage in renal cell carcinoma. Cancer Cell.

[21] Binnewies M., Roberts E.W., Kersten K., Chan V., Fearon D.F., Merad M., Coussens L.M., Gabrilovich D.I., Ostrand-Rosenberg S., Hedrick C.C. (2018). Understanding the tumor immune microenvironment (TIME) for effective therapy. Nat. Med..

[22] Hanahan D. (2022). Hallmarks of Cancer: New Dimensions. Cancer Discov..

[23] Luo W. (2023). Nasopharyngeal carcinoma ecology theory: Cancer as multidimensional spatiotemporal “unity of ecology and evolution” pathological ecosystem. Theranostics.

[24] Hinshaw D.C., Shevde L.A. (2019). The tumor microenvironment innately modulates cancer progression. Cancer Res..

[25] Vano Y., Giraldo N.A., Fridman W.H., Sautès-Fridman C. (2017). Oncoimmunology, A Practical Guide for Cancer Immunotherapy.

[26] Nixon B.G., Kuo F., Ji L., Liu M., Capistrano K., Do M., Franklin R.A., Wu X., Kansler E.R., Srivastava R.M. (2022). Tumor-associated macrophages expressing the transcription factor IRF8 promote T cell exhaustion in cancer. Immunity.

[27] Krishna C., DiNatale R.G., Kuo F., Srivastava R.M., Vuong L., Chowell D., Gupta S., Vanderbilt C., Purohit T.A., Liu M. (2021). Single-cell sequencing links multiregional immune landscapes and tissue-resident T cells in ccRCC to tumor topology and therapy efficacy. Cancer Cell.

[28] Shen H., Liu J., Chen S., Ma X., Ying Y., Li J., Wang W., Wang X., Xie L. (2021). Prognostic Value of Tumor-Associated Macrophages in Clear Cell Renal Cell Carcinoma: A Systematic Review and Meta-Analysis. Front. Oncol..

[29] Conejo-Garcia J.R., Rutkowski M.R., Cubillos-Ruiz J.R. (2016). State-of-the-art of regulatory dendritic cells in cancer. Pharmacol. Ther..

[30] Verneau J., Sautés-Fridman C., Sun C.-M. (2020). Dendritic cells in the tumor microenvironment: Prognostic and theranostic impact. Semin. Immunol..

[31] Collin M., Bigley V. (2018). Human dendritic cell subsets: An update. Immunology.

[32] Varn F.S., Wang Y., Mullins D.W., Fiering S., Cheng C. (2017). Systematic Pan-Cancer Analysis Reveals Immune Cell Interactions in the Tumor Microenvironment. Cancer Res..

[33] Wang T., Lu R., Kapur P., Jaiswal B.S., Hannan R., Zhang Z., Pedrosa I., Luke J.J., Zhang H., Goldstein L.D. (2018). An Empirical Approach Leveraging Tumorgrafts to Dissect the Tumor Microenvironment in Renal Cell Carcinoma Identifies Missing Link to Prognostic Inflammatory Factors. Cancer Discov..

[34] Braun D.A., Bakouny Z., Hirsch L., Flippot R., Van Allen E.M., Wu C.J., Choueiri T.K. (2021). Beyond conventional immune-checkpoint inhibition—Novel immunotherapies for renal cell carcinoma. Nat. Rev. Clin. Oncol..

[35] McRitchie B.R., Akkaya B. (2022). Exhaust the exhausters: Targeting regulatory t cells in the tumor microenvironment. Front. Immunol..

[36] Ahrends T., Borst J. (2018). The opposing roles of cd4+ t cells in anti-tumour immunity. Immunology.

[37] Turnis M.E., Sawant D.V., Szymczak-Workman A.L., Andrews L.P., Delgoffe G.M., Yano H., Beres A.J., Vogel P., Workman C.J., Vignali D.A. (2016). Interleukin-35 limits anti-tumor immunity. Immunity.

[38] Van der Leun A.M., Thommen D.S., Schumacher T.N. (2020). Cd8+ t cell states in human cancer: Insights from single-cell analysis. Nature reviews. Cancer.

[39] Fridman W.H., Meylan M., Petitprez F., Sun C.-M., Italiano A., Sautès-Fridman C. (2022). B cells and tertiary lymphoid structures as determinants of tumour immune contexture and clinical outcome. Nat. Rev. Clin. Oncol..

[40] Downs-Canner S.M., Meier J., Vincent B.G., Serody J.S. (2022). B Cell Function in the Tumor Microenvironment. Annu. Rev. Immunol..

[41] Lauss M., Donia M., Svane I.M., Jönsson G. (2022). B cells and tertiary lymphoid structures: Friends or foes in cancer immunotherapy?. Clin. Cancer Res..

[42] Iglesia M.D., Parker J.S., Hoadley K., Serody J.S., Perou C., Vincent B.G. (2016). Genomic Analysis of Immune Cell Infiltrates Across 11 Tumor Types. J. Natl. Cancer Inst..

[43] Murakami Y., Saito H., Shimizu S., Kono Y., Shishido Y., Miyatani K., Matsunaga T., Fukumoto Y., Ashida K., Sakabe T. (2019). Increased regulatory B cells are involved in immune evasion in patients with gastric cancer. Sci. Rep..

[44] Meylan M., Petitprez F., Becht E., Bougoüin A., Pupier G., Calvez A., Giglioli I., Verkarre V., Lacroix G., Verneau J. (2022). Tertiary lymphoid structures generate and propagate anti-tumor antibody-producing plasma cells in renal cell cancer. Immunity.

[45] Jonasch E., Walker C.L., Rathmell W.K. (2021). Clear cell renal cell carcinoma ontogeny and mechanisms of lethality. Nat. Rev. Nephrol..

[46] Becht E., Giraldo N.A., Lacroix L., Buttard B., Elarouci N., Petitprez F., Selves J., Laurent-Puig P., Sautes-Fridman C., Fridman W.H. (2016). Estimating the population abundance of tissue-infiltrating immune and stromal cell populations using gene expression. Genome Biol..

[47] Rooney M.S., Shukla S.A., Wu C.J., Getz G., Hacohen N. (2015). Molecular and Genetic Properties of Tumors Associated with Local Immune Cytolytic Activity. Cell.

[48] Şenbabaoğlu Y., Gejman R.S., Winer A.G., Liu M., Van Allen E.M., de Velasco G., Miao D., Ostrovnaya I., Drill E., Luna A. (2016). Tumor immune microenvironment characterization in clear cell renal cell carcinoma identifies prognostic and immunotherapeutically relevant messenger RNA signatures. Genome Biol..

[49] Ricketts C.J., Cubas A.A.D., Fan H., Smith C.C., Lang M., Reznik E., Bowlby R., Gibb E.A., Akbani R., Beroukhim R. (2018). The cancer genome atlas comprehensive molecular characterization of renal cell carcinoma. Cell Rep..

[50] Chevrier S., Levine J.H., Zanotelli V.R.T., Silina K., Schulz D., Bacac M., Ries C.H., Ailles L., Jewett M.A.S., Moch H. (2017). An Immune Atlas of Clear Cell Renal Cell Carcinoma. Cell.

[51] Li R., Ferdinand J.R., Loudon K.W., Bowyer G.S., Laidlaw S., Muyas F., Mamanova L., Neves J.B., Bolt L., Fasouli E.S. (2022). Mapping single-cell transcriptomes in the intra-tumoral and associated territories of kidney cancer. Cancer Cell.

[52] Calderaro J., Petitprez F., Becht E., Laurent A., Hirsch T.Z., Rousseau B., Luciani A., Amaddeo G., Derman J., Charpy C. (2019). Intra-tumoral tertiary lymphoid structures are associated with a low risk of early recurrence of hepatocellular carcinoma. J. Hepatol..

[53] Moussion C., Girard J.-P. (2011). Dendritic cells control lymphocyte entry to lymph nodes through high endothelial venules. Nature.

[54] Dieu-Nosjean M.-C., Goc J., Giraldo N.A., Sautès-Fridman C., Fridman W.H. (2014). Tertiary lymphoid structures in cancer and beyond. Trends Immunol..

[55] Goc J., Fridman W.-H., Sautès-Fridman C., Dieu-Nosjean M.-C. (2013). Characteristics of tertiary lymphoid structures in primary cancers. Oncoimmunology.

[56] Johansson-Percival A., He B., Li Z.-J., Kjellén A., Russell K., Li J., Larma I., Ganss R. (2017). De novo induction of intratumoral lymphoid structures and vessel normalization enhances immunotherapy in resistant tumors. Nat. Immunol..

[57] Johansson-Percival A., Ganss R. (2021). Therapeutic Induction of Tertiary Lymphoid Structures in Cancer Through Stromal Remodeling. Front. Immunol..

[58] Kazanietz M.G., Durando M., Cooke M. (2019). CXCL13 and Its Receptor CXCR5 in Cancer: Inflammation, Immune Response, and Beyond. Front. Endocrinol..

[59] Kroeger D.R., Milne K., Nelson B.H. (2016). Tumor-Infiltrating Plasma Cells Are Associated with Tertiary Lymphoid Structures, Cytolytic T-Cell Responses, and Superior Prognosis in Ovarian Cancer. Clin. Cancer Res..

[60] Sautès-Fridman C., Dimberg A., Verma V. (2022). Editorial: Tertiary Lymphoid Structures: From Basic Biology to Translational Impact in Cancer. Front. Immunol..

[61] Sautès-Fridman C., Petitprez F., Calderaro J., Fridman W.H. (2019). Tertiary lymphoid structures in the era of cancer immunotherapy. Nat. Rev. Cancer.

[62] Schumacher T.N., Thommen D.S. (2022). Tertiary lymphoid structures in cancer. Science.

[63] Vaghjiani R.G., Skitzki J.J. (2022). Tertiary Lymphoid Structures as Mediators of Immunotherapy Response. Cancers.

[64] Helmink B.A., Reddy S.M., Gao J., Zhang S., Basar R., Thakur R., Yizhak K., Sade-Feldman M., Blando J., Han G. (2020). B cells and tertiary lymphoid structures promote immunotherapy response. Nature.

[65] Clark D.J., Dhanasekaran S.M., Petralia F., Pan J., Song X., Hu Y., da Veiga Leprevost F., Reva B., Lih T.-S.M., Chang H.-Y. (2019). Integrated Proteogenomic Characterization of Clear Cell Renal Cell Carcinoma. Cell.

[66] Giraldo N.A., Becht E., Vano Y., Petitprez F., Lacroix L., Validire P., Sanchez-Salas R., Ingels A., Oudard S., Moatti A. (2017). Tumor-Infiltrating and Peripheral Blood T-cell Immunophenotypes Predict Early Relapse in Localized Clear Cell Renal Cell Carcinoma. Clin. Cancer Res..

[67] Hakimi A.A., Voss M.H., Kuo F., Sanchez A., Liu M., Nixon B.G., Vuong L., Ostrovnaya I., Chen Y.-B., Reuter V. (2019). Transcriptomic Profiling of the Tumor Microenvironment Reveals Distinct Subgroups of Clear Cell Renal Cell Cancer: Data from a Randomized Phase III Trial. Cancer Discov..

[68] Braun D.A., Hou Y., Bakouny Z., Ficial M., Angelo M.S., Forman J., Ross-Macdonald P., Berger A.C., Jegede O.A., Elagina L. (2020). Interplay of somatic alterations and immune infiltration modulates response to PD-1 blockade in advanced clear cell renal cell carcinoma. Nat. Med..

[69] Remark R., Alifano M., Cremer I., Lupo A., Dieu-Nosjean M.-C., Riquet M., Crozet L., Ouakrim H., Goc J., Cazes A. (2013). Characteristics and Clinical Impacts of the Immune Environments in Colorectal and Renal Cell Carcinoma Lung Metastases: Influence of Tumor Origin. Clin. Cancer Res..

[70] Bi K., He M.X., Bakouny Z., Kanodia A., Napolitano S., Wu J., Grimaldi G., Braun D.A., Cuoco M.S., Mayorga A. (2021). Tumor and immune reprogramming during immunotherapy in advanced renal cell carcinoma. Cancer Cell.

[71] Miao D., Margolis C.A., Gao W., Voss M.H., Li W., Martini D.J., Norton C., Bossé D., Wankowicz S.M., Cullen D. (2018). Genomic correlates of response to immune checkpoint therapies in clear cell renal cell carcinoma. Science.

[72] Samstein R.M., Lee C.-H., Shoushtari A.N., Hellmann M.D., Shen R., Janjigian Y.Y., Barron D.A., Zehir A., Jordan E.J., Omuro A. (2019). Tumor mutational load predicts survival after immunotherapy across multiple cancer types. Nat. Genet..

[73] Vuong L., Kotecha R.R., Voss M.H., Hakimi A.A. (2019). Tumor Microenvironment Dynamics in Clear-Cell Renal Cell Carcinoma. Cancer Discov..

[74] Turajlic S., Litchfield K., Xu H., Rosenthal R., McGranahan N., Reading J.L., Wong Y.N.S., Rowan A., Kanu N., Al Bakir M. (2017). Insertion-and-deletion-derived tumour-specific neoantigens and the immunogenic phenotype: A pan-cancer analysis. Lancet Oncol..

[75] Shapiro D.D., Virumbrales-Muñoz M., Beebe D.J., Abel E.J. (2022). Models of Renal Cell Carcinoma Used to Investigate Molecular Mechanisms and Develop New Therapeutics. Front. Oncol..

[76] Hsieh J.J., Purdue M.P., Signoretti S., Swanton C., Albiges L., Schmidinger M., Heng D.Y., Larkin J., Ficarra V. (2017). Renal cell carcinoma. Nat. Rev. Dis. Prim..

[77] Hoefflin R., Harlander S., Schäfer S., Metzger P., Kuo F., Schönenberger D., Adlesic M., Peighambari A., Seidel P., Chen C.-Y. (2020). HIF-1α and HIF-2α differently regulate tumour development and inflammation of clear cell renal cell carcinoma in mice. Nat. Commun..

[78] Xiong Y., Liu L., Xia Y., Qi Y., Chen Y., Chen L., Zhang P., Kong Y., Qu Y., Wang Z. (2019). Tumor infiltrating mast cells determine oncogenic HIF-2α-conferred immune evasion in clear cell renal cell carcinoma. Cancer Immunol. Immunother..

[79] Liu X.-D., Kong W., Peterson C.B., McGrail D.J., Hoang A., Zhang X., Lam T., Pilie P.G., Zhu H., Beckermann K.E. (2020). PBRM1 loss defines a nonimmunogenic tumor phenotype associated with checkpoint inhibitor resistance in renal carcinoma. Nat. Commun..

[80] McDermott D.F., Huseni M.A., Atkins M.B., Motzer R.J., Rini B.I., Escudier B., Fong L., Joseph R.W., Pal S.K., Reeves J.A. (2018). Clinical activity and molecular correlates of response to atezolizumab alone or in combination with bevacizumab versus sunitinib in renal cell carcinoma. Nat. Med..

[81] Carlisle J.W., Jansen C.S., Cardenas M.A., Sobierajska E., Reyes A.M., Greenwald R., Del Balzo L., Prokhnevska N., Kucuk O., Carthon B.C. (2022). Clinical outcome following checkpoint therapy in renal cell carcinoma is associated with a burst of activated CD8 T cells in blood. J. Immunother. Cancer.

[82] Giraldo N.A., Becht E., Pagès F., Skliris G.P., Verkarre V., Vano Y., Mejean A., Saint-Aubert N., Lacroix L., Natario I. (2015). Orchestration and Prognostic Significance of Immune Checkpoints in the Microenvironment of Primary and Metastatic Renal Cell Cancer. Clin. Cancer Res..

[83] Ghatalia P., Gordetsky J., Kuo F., Dulaimi E., Cai K.Q., Devarajan K., Bae S., Naik G., Chan T.A., Uzzo R. (2019). Prognostic impact of immune gene expression signature and tumor infiltrating immune cells in localized clear cell renal cell carcinoma. J. Immunother. Cancer.

[84] Cotta B.H., Choueiri T.K., Cieslik M., Ghatalia P., Mehra R., Morgan T.M., Palapattu G.S., Shuch B., Vaishampayan U., Van Allen E. Current Landscape of Genomic Biomarkers in Clear Cell Renal Cell Carcinoma. Eur. Urol..

[85] Motzer R.J., Robbins P.B., Powles T., Albiges L., Haanen J.B., Larkin J., Mu X.J., Ching K.A., Uemura M., Pal S.K. (2020). Avelumab plus axitinib versus sunitinib in advanced renal cell carcinoma: Biomarker analysis of the phase 3 JAVELIN Renal 101 trial. Nat. Med..

[86] Wallin J.J., Bendell J.C., Funke R., Sznol M., Korski K., Jones S., Hernandez G., Mier J., He X., Hodi F.S. (2016). Atezolizumab in combination with bevacizumab enhances antigen-specific T-cell migration in metastatic renal cell carcinoma. Nat. Commun..

[87] Motzer R.J., Banchereau R., Hamidi H., Powles T., McDermott D., Atkins M.B., Escudier B., Liu L.-F., Leng N., Abbas A.R. (2020). Molecular Subsets in Renal Cancer Determine Outcome to Checkpoint and Angiogenesis Blockade. Cancer Cell.

[88] Jansen C.S., Prokhnevska N., Master V.A., Sanda M.G., Carlisle J.W., Bilen M.A., Cardenas M., Wilkinson S., Lake R., Sowalsky A.G. (2019). An intra-tumoral niche maintains and differentiates stem-like CD8 T cells. Nature.

[89] Xu Y., Miller C.P., Warren E.H., Tykodi S.S. (2021). Current status of antigen-specific T-cell immunotherapy for advanced renal-cell carcinoma. Hum. Vaccines Immunother..

[90] Gopalakrishnan V., Spencer C.N., Nezi L., Reuben A., Andrews M.C., Karpinets T.V., Prieto P.A., Vicente D., Hoffman K., Wei S.C. (2018). Gut microbiome modulates response to anti–PD-1 immunotherapy in melanoma patients. Science.

[91] Routy B., le Chatelier E., DeRosa L., Duong C.P.M., Alou M.T., Daillère R., Fluckiger A., Messaoudene M., Rauber C., Roberti M.P. (2018). Gut microbiome influences efficacy of PD-1–based immunotherapy against epithelial tumors. Science.

[92] Derosa L., Hellmann M.D., Spaziano M., Halpenny D., Fidelle M., Rizvi H., Long N., Plodkowski A.J., Arbour K.C., Chaft J.E. (2018). Negative association of antibiotics on clinical activity of immune checkpoint inhibitors in patients with advanced renal cell and non-small-cell lung cancer. Ann. Oncol..

[93] Sivan A., Corrales L., Hubert N., Williams J.B., Aquino-Michaels K., Earley Z.M., Benyamin F.W., Lei Y.M., Jabri B., Alegre M.-L. (2015). Commensal Bifidobacterium promotes antitumor immunity and facilitates anti-PD-L1 efficacy. Science.

[94] Park E.M., Chelvanambi M., Bhutiani N., Kroemer G., Zitvogel L., Wargo J.A. (2022). Targeting the gut and tumor microbiota in cancer. Nat. Med..

[95] Dizman N., Meza L., Bergerot P., Alcantara M., Dorff T., Lyou Y., Frankel P., Cui Y., Mira V., Llamas M. (2022). Nivolumab plus ipilimumab with or without live bacterial supplementation in metastatic renal cell carcinoma: A randomized phase 1 trial. Nat. Med..

[96] Santoni M., Molina-Cerrillo J., Santoni G., Lam E.T., Massari F., Mollica V., Mazzaschi G., Rapoport B.L., Grande E., Buti S. (2023). Role of Clock Genes and Circadian Rhythm in Renal Cell Carcinoma: Recent Evidence and Therapeutic Consequences. Cancers.

